# Early-Life Circumstances and Late-Life Loneliness: Findings From In-Depth Interviews

**DOI:** 10.1093/geroni/igaa057.2061

**Published:** 2020-12-16

**Authors:** Elisa Tiilikainen

**Affiliations:** University of Eastern Finland, Kuopio, Finland

## Abstract

This case study examines loneliness from the perspective of two older men, who were interviewed three times as part of a five-year qualitative longitudinal study on loneliness in later life. Both interviewees self-identified as feeling lonely “often” or “all the time” and had experienced loneliness also in previous life phases. The interviews revealed trajectories of long-term loneliness which were impacted by life events and circumstances in early life, childhood and youth. Two critical experiences were identified: childhood bereavement and sexual abuse. These factors contributed to emotional insecurities and impacted the ways the interviewees perceived their selves and their relations with others. The acknowledgement of past life experiences is important for the theoretical and conceptual understanding of loneliness and the development of different intervention strategies. However, more longitudinal analysis is needed on the cumulative disadvantages making people vulnerable to long-term loneliness.

